# Effects of a diagnostic stewardship intervention to de-implement widespread use of a rapid respiratory multiplex PCR test

**DOI:** 10.1128/spectrum.00364-26

**Published:** 2026-07-28

**Authors:** Timothy C. Jenkins, Heather L. Young, David L. Wyles, Katherine C. Shihadeh, Allison L. Sabel, Read G. Pierce, Michael L. Wilson

**Affiliations:** 1 Division of Infectious Diseases, Denver Health 47804, Denver, Colorado, USA; 2 Division of Infectious Diseases, University of Colorado School of Medicine, Aurora, Colorado, USA; 3 Department of Pathology and Laboratory Services, Denver Health 47804, Denver, Colorado, USA; 4 Department of Pathology, University of Colorado School of Medicine, Aurora, Colorado, USA; 5 Department of Pharmacy, Denver Health 47804, Denver, Colorado, USA; 6 Department of Patient Safety and Quality, Denver Health 47804, Denver, Colorado, USA; 7 Department of Medicine, Denver Health 47804, Denver, Colorado, USA; 8 Department of Medicine, Division of Hospital Medicine, University of Colorado School of Medicine, Aurora, Colorado, USA; University of Mississippi Medical Center, Jackson, Mississippi, USA

**Keywords:** diagnostic stewardship, diagnostic excellence, rapid diagnostic testing

## Abstract

Rapid respiratory diagnostic tests are widely utilized but often do not change clinical management. We evaluated the effects of an intervention to de-implement widespread use of a multiplex respiratory pathogen PCR panel (RPP) in favor of a selective use strategy. This was a retrospective, quasi-experimental study conducted in a safety-net healthcare system. We compared RPP utilization during 2 years of unrestricted, widespread use (24 July 2022–23 July 2024) and 1 year after implementation of a selective use strategy (24 July 2024–23 July 2025). The primary outcome was change in weekly test volume as assessed by interrupted time-series analysis (ITS). During the widespread and selective use periods, 8,923 and 184 RPPs were performed, respectively. By ITS, test volume declined by 66 per week (*P* < 0.001) immediately after the intervention; this reduction was sustained over the subsequent year. Median weekly tests decreased from 83 (IQR 67–103) to 3 (IQR 2–5) (*P* < 0.001), a relative decline of 96.4%. The median age of patients tested decreased from 50 to 7 years (*P* < 0.001). The proportion of positive tests was similar (32.1% vs 35.9%; *P* = 0.28); however, the pathogens detected differed. Annualized laboratory costs decreased from $741,446 to $30,623. Use of an influenza/RSV/SARS-CoV-2 PCR decreased from a weekly median of 331 (IQR 189–586) to 118 (IQR 25–390) (*P* < 0.001). An intervention to de-implement widespread use of an RPP in favor of selective use led to a greater than 95% reduction in tests that was sustained over 1 year.

**IMPORTANCE:**

Use of rapid respiratory multiplex PCR panels (RPPs) is widespread but has not been shown to reduce antibiotic use or improve clinical outcomes. Reducing low-value use of these tests is therefore an important diagnostic stewardship target. To our knowledge, this is the first study describing a successful intervention to de-implement widespread use of this test among pediatric and adult patients and in ambulatory and inpatient care locations in favor of targeted use.

## INTRODUCTION

Use of rapid respiratory molecular diagnostic tests in the United States has increased dramatically in the last decade, particularly since the onset of the SARS-CoV-2 pandemic (1, 2). Such tests enable the timely identification of infectious organisms and facilitate early, appropriate treatment for certain respiratory pathogens, thus potentially improving patient outcomes. Rapid respiratory diagnostic tests also have important limitations, including the potential for false-positive results, identification of colonizing or appropriately treated organisms, and higher costs than traditional microbiological methods. These limitations are compounded when the tests are used in patients with a low pre-test probability of disease (3).

The BioFire Respiratory Panel (RP2.1) (BioFire Diagnostics, Salt Lake City, Utah, USA), hereafter referred to as the RPP, is a commonly used rapid respiratory molecular diagnostic test (4). This PCR-based, multiplex assay tests for 22 viral and bacterial targets from a single nasopharyngeal swab specimen in just over 1 h. Despite its relatively widespread use, the clinical utility of this test remains unclear. In a pilot randomized trial of adults and children presenting to an emergency department, randomization to use of the RPP as compared with usual care did not significantly lower the rate of antibacterial prescribing or increase the rate of antiviral prescribing and did not have an apparent effect on clinical outcomes (5). In a larger randomized trial of children with influenza-like illness presenting to an emergency department, clinician knowledge of RPP results was associated with a higher frequency of antibacterial prescribing and did not affect the frequency of antiviral prescribing (6). Additional randomized trials of similar respiratory multiplex PCR panels in adult inpatient and pediatric emergency departments did not reduce use of antibiotics or affect length of hospital stay (7, 8).

We observed a substantial increase in RPP utilization across our organization during the SARS-CoV-2 pandemic; this persisted even after SARS-CoV-2 testing recommendations were relaxed. An internal review of test utilization demonstrated frequent testing of low-risk patients in ambulatory care settings, little apparent change in clinical decision-making in both ambulatory and inpatient settings, and a large cost to the microbiology laboratory. For these reasons, our organization opted to discontinue widespread use of this test in favor of a selective use strategy. The objectives of this study were to evaluate the effects of this intervention on RPP utilization, characteristics of patients tested, and test results.

## MATERIALS AND METHODS

### Study setting

Our institution is an academic, integrated, safety-net healthcare system consisting of a 555-bed acute care hospital, an emergency department, four urgent care centers, 10 community health centers, 20 school-based health clinics, and specialty clinics. The health system cares for nearly 25% of the surrounding county’s population annually.

The initial version of the BioFire Respiratory Panel included 17 viral targets (adenovirus, coronaviruses 229E, HKU1, NL63, and OC43, human metapneumovirus, human rhinovirus/enterovirus, influenza A with subtypes H1, H3, and H1-2009, influenza B, parainfluenza viruses 1–4, and respiratory syncytial virus [RSV]) and three bacterial targets (*Bordetella pertussis*, *Mycoplasma pneumoniae*, and *Chlamydia pneumoniae*). This test was first implemented at our institution in October 2013. The BioFire Respiratory Panel (RP2.1) with added targets for SARS-CoV-2 and *Bordetella parapertussis* was implemented in December 2020. Both tests were available for use in all ambulatory and inpatient settings without restriction; there were no standardized criteria for use. The estimated cost to the laboratory for the RPP was $166.43 per reportable test result.

### Intervention

A multidisciplinary committee reviewed data on utilization patterns of the RPP, results, and clinical cases in which the test was used and determined that an intervention was warranted to discontinue widespread, routine use of the test in favor of a selective use strategy. The selective strategy included a recommendation to test individuals for treatable viruses (influenza and SARS-CoV-2) using an influenza A/B, RSV, and SARS-CoV-2 PCR panel (Xpert Xpress CoV-2/Flu/RSV Plus; Cepheid, Sunnyvale, California, USA) only if a positive result would lead to antiviral treatment. The RPP was recommended for use only when influenza, RSV, and SARS-CoV-2 were not detected on the targeted panel and when (i) it was felt that detection of one of the other pathogens on the RPP would influence clinical decision-making, or (ii) diagnostic certainty of the infecting pathogen was considered essential to guide management. The rationale and plan for the intervention were pre-communicated initially to department heads and then to all providers, pharmacists, and nurses prior to implementation of the selective use strategy. The following approaches were used to implement the selective use strategy: (i) the order for the RPP was removed from the electronic health record (Epic; Verona, Wisconsin) at the time of intervention implementation on 24 July 2024 (ii); Infectious Diseases attending approval was required for the RPP to be performed for the first 3–4 months of the intervention (iii); and based on the pattern of RPP requests and approvals during this initial period, select groups of attending physicians in the acute care setting (medical ICU, inpatient pediatrics, and pediatric emergency department) were subsequently provided access to order the test.

The influenza A/B, RSV, and SARS-CoV-2 PCR panel and an individual SARS-CoV-2 PCR (Cepheid Xpert Xpress CoV-2 plus) were available for use without restriction before and after the intervention. A *B. pertussis/parapertussis* PCR (DiaSorin) was available beginning in November 2022, and the BioFire Pneumonia multiplex PCR panel was available only in intensive care units beginning in July 2023.

### Study design

We conducted a retrospective quasi-experimental study of all patients in whom the RPP was performed during the two years prior to the intervention when use of the test was widespread (24 July 2022–23 July 2024), and for 1 year after implementation of the intervention (i.e., selective use strategy) (24 July 2024–23 July 2025).

### Outcomes

The primary outcome was the weekly number of RPPs performed before and after the intervention, assessed by interrupted time series analysis. Secondary outcomes included the median number of tests per week, characteristics and locations of patients tested, the percentage of positive tests, the frequency of individual RPP targets identified, laboratory costs, and alternative rapid respiratory tests performed.

### Statistical analysis

All statistical analyses were performed using SAS Enterprise Guide (version 8.3). Interrupted time series analysis was performed using PROC AUTOREG. The Durbin-Watson statistic was used to identify autocorrelation; however, autocorrelation was resolved with two lags (DW = 1.95, *P* = 0.31). To evaluate differences in median values and categorical variables between the pre-intervention and intervention periods, the Mann-Whitney *U* test and χ^2^ (or Fisher’s exact) test were used, respectively. A *P* value of < 0.05 was considered statistically significant. An institutional Quality Improvement Review Committee, authorized by the Colorado Multiple Institutional Review Board, reviewed the study proposal and determined that it was not human subjects research. Data from this study are available upon request.

## RESULTS

**Fig 1:**
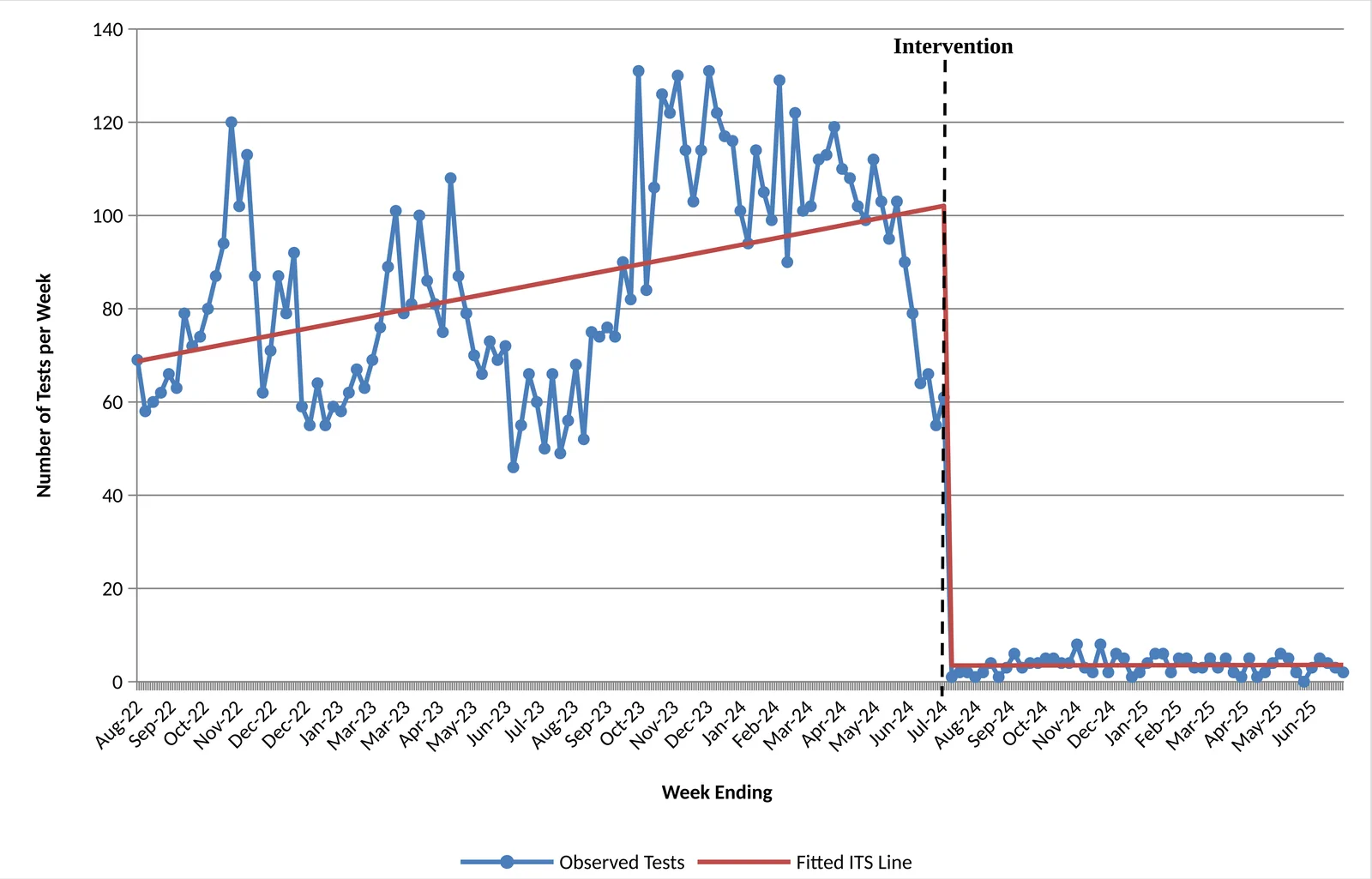
Interrupted time series analysis (ITS) of RPP utilization over time

During the two-year pre-intervention period of unrestricted use, a total of 8,923 RPPs were performed; 184 RPPs were performed during the 1 year selective use period (Table 1). Prior to the intervention, there was a non-significant increase in utilization RPP of 0.16 per week (*P* = 0.39) (Fig. 1; Table S1). Immediately after implementing the selective use strategy, there was a significant decrease of 66 tests per week (*P* < 0.001). This change was sustained over the intervention period without a significant change in the post-intervention slope of testing.

**TABLE 1:** Test volume, results, and characteristics of patients tested^*c*^

	Pre-intervention period 7/24/22–7/23/24 *N* = 8,923	Selective use period 7/24/24–7/23/25 *N* = 184	*P* value
Test performed per week, median (IQR)	83 (67–103)	3 (2–5)	<0.001
Patient age, median, IQR, range	50 (29-64), 0-99	7.5 (0–41), 0–87	<0.001
<1 year	632 (7.1)	69 (37.5)	
1–18 years	792 (8.9)	42 (22.8)	
19–64 years	5315 (59.6)	60 (32.6)	
≥65 years	2182 (24.5)	13 (7.1)	
Adult (age >18 years), *n* (%)	7497 (84.0)	73 (39.7)	<0.001
Female, *n* (%)	4058 (45.5)	85 (46.2)	0.92
Race, *n* (%)			<0.001
White	5051 (56.6)	87 (47.3)	
Black or African American	1230 (13.8)	15 (8.2)	
Asian	217 (2.4)	7 (3.8)	
Two or more races	413 (4.6)	8 (4.4)	
Other	1957 (21.9)	64 (34.8)	
Unknown or did not answer	55 (0.6)	3 (1.6)	
Ethnicity, *n* (%)			0.002
Hispanic or Latino	4105 (46.0)	108 (58.7)	
Not Hispanic or Latino	4755 (53.3)	76 (41.3)	
Unknown or did not answer	63 (0.7)	0 (0.0)	
Primary language, *n* (%)			<0.001
English	6711 (75.2)	107 (58.2)	
Spanish	1884 (21.1)	61 (33.2)	
Other	328 (3.7)	16 (8.7)	
Clinical location, *n* (%)			<0.001
Outpatient clinic	605 (6.8)	5 (2.7)	
Adult ED or urgent care	5165 (57.9)	12 (6.5)	
Pediatric ED or urgent care	912 (10.2)	51 (27.7)	
Inpatient non-ICU unit	1522 (17.1)	56 (30.4)	
Adult intensive care unit	444 (5.0)	27 (14.7)	
PICU/NICU	85 (1.0)	31 (16.9)	
OB/GYN unit	166 (1.9)	2 (1.1)	
Other	24 (0.3)	0 (0.0)	
Positive test (any target detected), *n* (%)	2864 (32.1)	66 (35.9)	0.28
Total targets detected^*a*^	3159	76	
Rhinovirus/enterovirus	1163 (36.8)	30 (39.5)	
Parainfluenza	298 (9.4)	12 (15.8)	
Influenza A or B	308 (9.7)	6 (7.9)	
SARS-CoV-2	514 (16.3)	2 (2.6)	
Adenovirus	166 (5.3)	4 (5.3)	
Human metapneumovirus	205 (6.5)	7 (9.2)	
Coronavirus	274 (8.7)	4 (5.3)	
RSV	215 (6.8)	3 (3.9)	
*M. pneumoniae*	9 (0.3)	8 (10.5)	
*B. pertussis/parapertussis*	7 (0.2)	0	
*Chlamydia pneumoniae*	0	0	
Total laboratory test costs, annualized $^*b*^	$741,446	$30,623	
Inpatient tests	$170,508	$18,973	
Outpatient tests	$570,938	$11,650	
Alternative rapid respiratory tests performed per week, median (IQR)			
Influenza A/B, RSV, and SARS-CoV-2 PCR	331 (189–586)	118 (25–390)	<0.001
SARS-CoV-2 PCR	68 (48–116)	27 (9–103)	<0.001
BioFire Pneumonia panel	4 (3–6)	6 (4–8)	0.01
*B. pertussis/parapertussis* PCR	2 (1–3)	7 (3–11)	<0.001

^*a*^ Individual panel targets reported as *n* (% of total targets detected).

^*b*^ Based on an estimated cost per reportable of $166.43 US dollars.

^*c*^ IQR, interquartile range; ED, emergency department.

The median number of tests performed per week decreased from 83 (interquartile range [IQR] 67–103) prior to the intervention to 3 (IQR 2–5) (*P* < 0.001) during the selective use period, a relative reduction of 96% (Table 1). The median age of patients tested declined significantly from 50 years (IQR 29–64) to 7 years (IQR 0–41) (*P* < 0.001). Approximately 60% of patients tested during the selective use period were children compared with 16% prior to the intervention (*P* < 0.001). The intervention was also associated with a significant shift in clinical locations where testing was performed (Table 1). Prior to the intervention, most RPPs were performed in the adult emergency department (57.8%), whereas after the intervention they were concentrated in hospital inpatient units and the pediatric emergency department. The clinical rationale for RPP requests during the selective use period included the following general themes. A positive result could (1) avert additional and potentially invasive diagnostic tests; (2) influence antibiotic treatment decisions for atypical bacterial targets on the panel; (3) influence non-antibiotic treatment decisions; and (4) influence infection prevention decisions. Additional details of clinical rationales are provided in Table 2.

**TABLE 2:** Themes and examples of clinician rationales for RPP testing during the selective use period

Themes	Specific examples
Identification of a pathogen on the RPP could prevent the need for additional diagnostic testing or an invasive procedure	Neonates and infants presenting to the pediatric ED (or who are hospitalized) with fever to avoid a lumbar puncture
	Adults in the medical ICU with suspected viral illness and acute respiratory failure (but not intubated) to avoid need for high-risk bronchoscopy
	Concern for infectious myocarditis, where pathogen identification could prevent the need for a myocardial biopsy
	Evaluation of etiology of transverse myelitis
Identification of a pathogen on the RPP could provide prognostic information or influence decision-making regarding non-antibiotic therapy	An adult with severe exacerbation of chronic lung disease, where identification of human metapneumovirus would portend a protracted illness and lead the pulmonologist to extend the duration of the corticosteroid taper
	Pediatric patient with Kawasaki-like syndrome, where the presence or absence of adenovirus may influence the decision to initiate intravenous immune globulin
Clinical suspicion for atypical bacterial pathogen on the panel	A pediatric patient with suspected reactive infectious mucocutaneous eruption (RIME), where the RPP was used to confirm the etiology due to *M. pneumoniae*
	Prolonged respiratory illnesses in school-aged children with suspicion for *M. pneumoniae*
	Post-tussive emesis with clinical suspicion for *B. pertussis* (a specific Bordetella PCR would likely be a more appropriate test)
	Confirmed etiology of aseptic meningitis (*M. pneumoniae*) in an adolescent hospitalized with aseptic meningitis after a recent prolonged respiratory illness
Infection prevention decision-making	Evaluation of suspected nosocomial transmission of a pathogen on the RPP
	Identification of a pathogen on the RPP may influence infection prevention decisions for high-risk neonates in the ICU

The proportion of RPPs with at least one target detected did not differ significantly between periods (32.0% vs 35.9%; *P* = 0.26) (Table 1). Detections of influenza, RSV, or SARS-CoV-2 declined (32.8% of targets detected vs 14.5%, *P* < 0.001), whereas detections of other viruses on the panel (66.7% vs 75.0%, *P* = 0.09) and *Mycoplasma pneumoniae* (0.3% vs 10.5%, *P* < 0.001) were more frequent.

Annualized laboratory costs for RPP testing decreased from $741,446 during the unrestricted period to $30,623 during the selective use period (Table 1). Overall utilization of the influenza A/B, RSV, and SARS-CoV-2 PCR panel and the individual SARS-CoV-2 PCR decreased during the intervention period while use of the BioFire Pneumonia Panel and *B. pertussis/parapertussis* PCR modestly increased (Table 1).

## DISCUSSION

An intervention to de-implement widespread use of the RPP in favor of a selective use strategy led to a greater than 95% reduction in tests performed over the first year. The location of test utilization shifted from the adult emergency department and other ambulatory care locations to hospital inpatient units during the selective use period. In addition, children were more frequently tested than adults after the intervention.

There have been prior reports of diagnostic stewardship interventions to reduce potentially low-value use of RPPs in children with respiratory infections (9, 10). To our knowledge, this is the first description of an intervention to reduce widespread use of RPPs spanning adult and pediatric populations and ambulatory and inpatient care locations. During the period of widespread use, the large volume of RPPs was driven primarily by utilization in the adult emergency department and urgent care centers, as well as non-ICU inpatient units. Once the selective use strategy was implemented, requests from these clinical locations were infrequent, suggesting the test had not been adding substantial diagnostic or clinical value. In contrast, requests for the test during the selective use period were most commonly received from the medical ICU, neonatal ICU, pediatric ICU, pediatric acute care unit, and pediatric emergency department. The rationale for these test requests was often not to influence the decision to prescribe antibiotics, but to potentially prevent the need for other diagnostic tests or to inform non-antibiotic therapeutics. These requests highlight that although randomized trials have not shown use of the RPP to be associated with substantial changes in antibiotic utilization, the test may still have importance for clinical decision-making or diagnostic certainty in a subset of cases. Thus, diagnostic stewardship efforts around use of this test must consider more than just its effects on antibiotic use. This finding also highlights the importance of incorporating outcomes such as procedures or diagnostic tests averted and changes in non-antibiotic utilization in future trials.

It is worth reiterating that our strategy to reduce widespread use of the RPP included a recommendation to evaluate for ‘treatable viruses’ using a more targeted influenza/RSV/SARS-CoV-2 PCR panel. Although we believe this was a more clinically appropriate and cost-effective test in most scenarios, particularly in the ambulatory care setting, this diagnostic strategy had the potential to increase use of the influenza/RSV/SARS-CoV-2 PCR panel or other alternative respiratory tests. In contrast, during the intervention period, we observed an overall decrease in influenza/RSV/SARS-CoV-2 PCR panels and in individual SARS-CoV-2 PCR tests and a mild increase in tests for pertussis. Although the details are outside the scope of this report, we attribute these declines at least in part to additional institutional diagnostic stewardship efforts to promote more clinically appropriate use of these tests. This included education and a communication campaign with two primary messages: (i) testing is not indicated to inform outpatient isolation practices given national guidelines now recommend the same isolation approach regardless of the type of respiratory virus (11), and (ii) use of the influenza/RSV/SARS-CoV-2 PCR panel should be prioritized in patients who would be prescribed an antiviral agent if they tested positive for influenza or SARS-CoV-2. Furthermore, use of the influenza/RSV/SARS-CoV-2 PCR panel was discouraged outside of respiratory season by temporary removal of the order from the electronic health record during the summer months of 2025.

Our approach to achieving selective test utilization warrants additional discussion. After an initial period during which approval from an Infectious Diseases attending physician was required, we transitioned to a unique strategy of allowing attending physicians in certain acute care locations to order the test. Since we are a teaching institution, one could argue these attending-only orders reduced the autonomy and mastery of our learners (students, residents, and fellows), who are key targets for acquiring high-value practices during their training. The rationale for this approach included the following. First, learners who rotate at our hospital also rotate at affiliated hospitals where the RPP was widely used without restriction. We therefore believed it would be extremely difficult to change ordering patterns of learners at our hospital alone. Although a coordinated effort to change testing practices among affiliated hospitals would have been optimal, this was not practical, feasible, or within the control of the authors at the time of this work. Second, attending physicians from multiple clinical locations independently suggested this approach as a potential method to ensure that learners discussed the need for an RPP with an attending physician, and by virtue of placing the order, that the attending agreed the test was clinically appropriate. Interestingly, we did not observe an increase in tests performed after transitioning to the attending-only order strategy, indicating that this is a potentially effective approach to promote selective use of diagnostic tests which may obviate the need for an approval process that relies on the Infectious Diseases service or microbiology laboratory. Whether this approach is effective over time is unclear; however, we hope to continue to evolve the ordering approach to balance provider autonomy and trainee learning with the need for selective use of this test.

Although the proportion of RPPs that was positive before and after the intervention was similar, we observed differences in the distribution of pathogens. The fact that influenza, RSV, and SARS-CoV-2 accounted for a smaller proportion of targets detected was not unexpected given the RPP was intended to be used only after these pathogens had been excluded by the more targeted test. Conversely, other targets were detected in a larger proportion of cases with the selective use strategy; although the absolute number of cases was small, the relative increase in *M. pneumoniae* detections was notable. This could be due to a national increase in mycoplasma infections noted during 2024 (12), an increase in the proportion of RPPs utilized in children with signs and symptoms potentially consistent with mycoplasma infection, or both. The facilitation of the appropriate treatment of *M. pneumoniae* represents a potential benefit of RPP use in select clinical scenarios.

The Centers for Disease Control and Prevention Core Elements of Diagnostic Excellence state that diagnostic excellence involves not only making accurate and timely diagnoses but maximizing patient satisfaction and managing diagnostic uncertainty (13). We did not evaluate changes in satisfaction metrics for clinical locations such as the emergency department and urgent care given concern that these metrics may not be sensitive enough to detect potential changes in satisfaction among patients presenting with a respiratory illness in whom a specific etiology of the illness was not discovered. Thus, further evaluation of the effects of selective RPP utilization on patient satisfaction is warranted. Similarly, the effects of the marked reduction in use of this test on providers warrant further consideration. For example, did the selective use strategy lead to a problematic increase in diagnostic uncertainty or difficult patient interactions due to the lack of the test’s widespread availability? It is notable that the test was commonly used in the adult emergency department, urgent care, and outpatient clinics before the intervention but was infrequently requested in these locations after the intervention (total of 17 tests). This suggests that despite the widespread use when unrestricted, it may not have been providing substantial incremental clinical benefit over more targeted viral testing. This would seem to be consistent with the randomized trials discussed above that did not show substantial changes in management associated with use of this test.

This study has a number of limitations. First, the intervention was conducted in a single, academic, integrated healthcare system, which limits its generalizability. Second, although the intervention was strongly associated with a reduction in the number of tests performed, this study did not seek to answer directly whether the appropriateness of test utilization was affected. Third, although changes in test utilization were maintained over a 1 year period, whether they can be sustained over a longer period requires further study. Fourth, the effects of this large change in test utilization on clinical outcomes are not known; however, as discussed, prior randomized trials have not demonstrated improved outcomes with use of this test, and our internal safety event monitoring system did not suggest clinician concerns for a decrement in quality of care. Providers also continued to test for treatable viruses; thus, one would not expect a difference in outcomes. Finally, we present only changes in laboratory costs of the RPP associated with the intervention. This is an oversimplification as it does not take into account reimbursement for RPP tests performed or changes in utilization of alternative respiratory tests. Despite these limitations, this study highlights the opportunity to promote higher value care through selective use of a test that has not been clearly shown to directly influence treatment decisions or improve outcomes.

In conclusion, de-implementation of widespread use of the RPP across an academic safety-net organization in favor of a selective use strategy after excluding influenza, SARS-CoV-2, and RSV led to an immediate and marked reduction in test utilization that was sustained over 1 year. Utilization shifted toward acute care settings and became more concentrated among pediatric patients. An attending-only order approach was effective to maintain selective use of this test. The sustainability of this approach and its acceptability to patients and providers require further study.

## SUPPLEMENTAL MATERIAL

Table S1 spectrum.00364-26-s0001.docx Interrupted time series analysis of RPP utilization over time.

ASM does not own the copyrights to Supplemental Material that may be linked to, or accessed through, an article. The authors have granted ASM a non-exclusive, world-wide license to publish the Supplemental Material files. Please contact the corresponding author directly for reuse.
