# Supplementary material for: Effects of a diagnostic stewardship intervention to de-implement widespread use of a rapid respiratory multiplex PCR test
Source: Microbiol Spectr. 2026 Jul 28;14(9):e00364-26. doi: 10.1128/spectrum.00364-26 (PMC13532351; doi:10.1128/spectrum.00364-26)
Supplement: Supplementary file 1 [file spectrum.00364-26-s0001.docx]

**Supplement**

**Effects of a Diagnostic Stewardship Intervention to De-implement Widespread Use of a Rapid Respiratory Multiplex PCR Test**

**Supplemental Table.** Interrupted time series analysis of RPP utilization over time

| **Term** | **Coefficient** | **95% CI** | **Std. Error** | **P-value** |
| --- | --- | --- | --- | --- |
| Intercept (baseline level) | 73.02 | 48.89 to 97.15 | 12.31 | <0.001 |
| Pre-intervention slope (per week) | 0.16 | -0.21 to 0.54 | 0.19 | 0.39 |
| Immediate level change at intervention | -65.99 | -85.09 to -46.89 | 9.75 | <0.001 |
| Change in slope after intervention | -0.66 | -1.69 to 0.37 | 0.53 | 0.21 |
